# Design, evaluation, and immune simulation of potentially universal multi-epitope mpox vaccine candidate: focus on DNA vaccine

**DOI:** 10.3389/fmicb.2023.1203355

**Published:** 2023-07-21

**Authors:** Nino Rcheulishvili, Jiawei Mao, Dimitri Papukashvili, Shunping Feng, Cong Liu, Xingyun Wang, Yunjiao He, Peng George Wang

**Affiliations:** Department of Pharmacology, School of Medicine, Southern University of Science and Technology, Shenzhen, China

**Keywords:** monkeypox, mpox virus, universal vaccine, DNA vaccine, immunoinformatics, *in silico*, epidemics, pandemics

## Abstract

Monkeypox (mpox) is a zoonotic infectious disease caused by the mpox virus. Mpox symptoms are similar to smallpox with less severity and lower mortality. As yet mpox virus is not characterized by as high transmissibility as some severe acute respiratory syndrome 2 (SARS-CoV-2) variants, still, it is spreading, especially among men who have sex with men (MSM). Thus, taking preventive measures, such as vaccination, is highly recommended. While the smallpox vaccine has demonstrated considerable efficacy against the mpox virus due to the antigenic similarities, the development of a universal anti-mpox vaccine remains a necessary pursuit. Recently, nucleic acid vaccines have garnered special attention owing to their numerous advantages compared to traditional vaccines. Importantly, DNA vaccines have certain advantages over mRNA vaccines. In this study, a potentially universal DNA vaccine candidate against mpox based on conserved epitopes was designed and its efficacy was evaluated *via* an immunoinformatics approach. The vaccine candidate demonstrated potent humoral and cellular immune responses *in silico*, indicating the potential efficacy *in vivo* and the need for further research.

## 1. Introduction

The history of pandemics has shown us that not a single virus outbreak should be neglected as sooner or later the re-emergence of the same or even more virulent strain may occur with severe outcomes. The spread of the monkeypox (mpox) virus was considered limited to Central and West African countries until May 2022 when it crossed the continental borders and gained global health emergency status by World Health Organization (WHO) on 23 July 2022 (Papukashvili et al., 2022b; World Health Organization, 2022b). Mpox is a zoonotic disease with similar clinical manifestation as the smallpox virus which also belongs to the genus *Orthopoxvirus* with an additional symptom of lymph node enlargement. Mpox disease is characterized by a rash appearing 1–3 days following the onset of fever and lymphadenopathy. The rash is usually distributed on the whole body, especially on extremities (Sklenovská and Van Ranst, 2018), genitals, and anus (Kmiec and Kirchhoff, 2022; Mileto et al., 2022; Moschese et al., 2022; Sah et al., 2022; World Health Organization, 2022a). The mpox virus is a large, ~280 nm X ~220, brick/oval-shaped virus with a large linear double-stranded ~197 kb long DNA genome (Papukashvili et al., 2022b) with more than 190 open reading frames (ORFs) (Shchelkunov et al., 2002; Kugelman et al., 2014; Kmiec and Kirchhoff, 2022; Vandenbogaert et al., 2022; Zhu et al., 2022). The mpox virus produces two infectious forms of virions from the infected cells—an intracellular mature virion (IMV) and an extracellular enveloped virion (EEV) with an extra envelope. The structure of the mpox virus is complex, and many of the viral antigens are not well-studied. Infection by EEV is more efficient than by IMV (Locker et al., 2000).

One of the main reasons for the recent mpox multi-country outbreaks is the cessation of smallpox vaccination in 1980 which seems reasonable as most of the current mpox cases are detected in smallpox-unvaccinated people (Papukashvili et al., 2022b). Although the smallpox vaccine which is based on the vaccinia virus, another representative of *Orthopoxvirus*, is effective against the mpox virus (Zandi et al., 2023), there is no specific mpox vaccine available till now. The modified vaccinia virus Ankara-Bavarian Nordic (MVA-BN), also known as Imvamune, Jynneos, or Imvanex, is a third-generation authorized smallpox vaccine and is also used as the mpox vaccine (Zaeck et al., 2022). Although the currently available vaccine is effective for the mpox virus (CDC, 2022; Kandeel et al., 2023), Zaeck et al. have demonstrated that immunization series with the two-shot MVA-BN vaccine, in non-primed individuals, yields relatively low levels of mpox virus-neutralizing antibodies (Zaeck et al., 2022). The line of preclinical studies has made efforts in the development of the mpox vaccine. Hooper et al. have shown that the DNA vaccine encoding the antigens L1, A27, A33, and B5R protected rhesus macaques from the lethal challenge of the mpox virus (Hooper et al., 2004). Other studies have provided evidence of protection from the mpox virus challenge in non-human primates after immunization with A27, A33, B5, and L1 proteins (Buchman et al., 2010; Heraud et al., 2022). However, these vaccines were strain-specific. Importantly, recently worldwide spread mpox virus is of the West African clade with a mortality rate of approximately 1–3.6% (Kmiec and Kirchhoff, 2022; Kozlov, 2022; Yang, 2022), while the mpox of Central African clade is deadlier with a mortality rate of up to 11% (Jezek et al., 1987; Shafaati and Zandi, 2022; Yang, 2022). Considering the possible severe outcomes of whether the mpox virus of the Central African clade is spread, developing a new universal vaccine against mpox is urgently needed. Nucleic acid vaccines have gained enormous attention in recent years due to their efficacy, safety, cost-effectiveness, and time-saving features compared with conventional approaches. Apparently, the available mRNA vaccines for the current coronavirus disease 2019 (COVID-19) have high efficacy and safety profiles (Pardi et al., 2018; Corbett et al., 2020; Walsh et al., 2020; Haas et al., 2021; Kowalzik et al., 2021; Liu et al., 2022b). On the other hand, studies on DNA vaccines demonstrated no less advantageous features than mRNA vaccines including the requirement of even less production time and high stability (Flingai et al., 2013; Williams, 2013; Chavda et al., 2021b; Xia et al., 2021; Wang et al., 2022). More importantly, there is already an available DNA vaccine for COVID-19 approved in India (Sheridan, 2021). According to the current situation of available vaccines and the general epidemiological picture, developing a universal next-generation effective anti-mpox vaccine is necessary. To design a potentially universal DNA vaccine contender against mpox virus, in the present research, according to their functions and immunogenicity (Shchelkunov et al., 2002; Hooper et al., 2004; Buchman et al., 2010; Hirao et al., 2011; Meng et al., 2018; Zhang et al., 2023), sequences of four antigen proteins (A5L, A15L, A35R, and B6R) were retrieved and aligned, and the conserved sequences were selected to predict B-cell, MHC-I, and MHC-II binding epitopes. Except for the importance of immune response and protection against viral challenges, another rationale behind choosing these representative antigens was to assess the effectiveness of the novel antigen combination, aiming to propose another candidate for an anti-mpox vaccine. After the design of the vaccine, the final construct was optimized, and the structure and various characteristics of the vaccine were predicted. Immune simulation analyses demonstrated the strong humoral and cellular responses that warrant the contribution of the vaccine candidate in potentially universal multi-epitope DNA vaccine development.

## 2. Materials and methods

### 2.1. Selection of antigens, collection of data, and selection of conserved sequences

Four antigen proteins, namely, A5L, A15L, A35R, and B6R, were chosen according to their function in the life cycle and immunogenicity of the mpox virus. This selection also aimed to assess the immune responses generated by the vaccine against this combination of antigens. They are the orthologs of vaccinia virus glycoproteins—A4L, A14L, A33R, and B5R, respectively (Shchelkunov et al., 2002). These antigens are highly conserved among the mpox, vaccinia, and variola viruses. The protein sequences of mpox virus antigens reported on the National Center for Biotechnology Information (NCBI) database were retrieved. The number of downloaded sequences for A5L, A15L, A35R, and B6R was 1819, 355, 349, and 376, respectively. All the sequences of A5L were used for further analyses as they met the selection criteria such as the known length of the protein sequence. For the rest of the three proteins, the redundant sequences were removed according to their length and quality−179 sequences of A15L, 178 sequences of A35R, and 178 sequences of B6R were used for further analyses. The software Jalview 2.11.1.4 was used for the alignment and conservancy analysis (Waterhouse et al., 2009). The threshold filter of conservation was adjusted to 10 below the threshold, and the consensus sequence was used for B-cell and T-cell (MHC-I and MHC-II binding) epitope prediction.

### 2.2. Epitope prediction and selection

NetMHCpan EL 4.1 server was applied for the prediction of cytotoxic T lymphocyte (CTL) epitopes (https://services.healthtech.dtu.dk/service.php?NetMHCpan-4.1) (Reynisson et al., 2020). This server predicts the binding of peptides to any MHC-I molecule of the known sequence using artificial neural networks (ANNs) that are a collection of simple interconnected algorithms processing information according to the external input. The globally most prevalent alleles HLA-A^*^01:01, HLA-B^*^07:02, and HLA-B^*^08:01 were selected from the “Allele Frequency Net Database” and used for the prediction of the MHC-I binding epitope. The default parameters of the server were used: the threshold for strong binders was set to 0.5 (% rank), while for weak binders it was 2%. The predicted peptides were identified as strong binders when the % rank was below the set threshold (0.5%), while the weak binders were considered when the peptide rank was above 0.5% and below 2%. For the prediction of helper T lymphocyte (HTL) epitopes, the NetMHCIIpan-4.0 server which performs the prediction of peptide binding to any MHC-II molecule of the known sequence using ANNs was used. The worldwide most prevalent MHC-II alleles DRB1^*^07:01 and DRB1^*^15:01 were selected for the prediction of MHC-II binding peptides of the mpox virus conserved sequences. According to the default parameters, the predicted peptides were identified as strong binders when the % rank was below the set threshold (1%), while the weak binders were considered when the peptide rank was above 1% and below 2%. The epitopes with the strongest binding capacity to MHC-I and MHC-II alleles were selected. Both NetMHCpan-4.1 and NetMHCIIpan-4.0 use the NNAlign_MA machine learning framework, which has ANNs at its core. NetMHCpan-4.1, a computational tool, has been trained using a comprehensive dataset comprising over 850,000 peptides with quantitative binding affinity (BA) and mass spectrometry-eluted ligand (EL) information. The BA data utilized in the training of NetMHCpan-4.1 encompass 170 MHC molecules from various species—humans (HLA-A, HLA-B, HLA-C, and HLA-E), mice (H-2), cattle (BoLA), primates (Patr, Mamu, and Gogo), equine (Eqca), and swine (SLA). The EL data used in the analysis include 177 MHC molecules from a range of species, such as humans (HLA-A, B, C, E), mice (H-2), cattle (BoLA), primates (Patr, Mamu, and Gogo), swine (SLA), equine (Eqca), and dogs (DLA). NetMHCIIpan-4.0 has been trained using a comprehensive dataset comprising more than 500,000 measurements of BA and EL. This dataset covers the three human MHC class II isotypes, namely HLA-DR, HLA-DQ, and HLA-DP, along with the mouse molecules (H-2). The inclusion of EL data expands the coverage of MHC-II molecules, as the BA data cover 59 molecules, while the EL data cover 74 molecules. Both NetMHCpan-4.1 and NetMHCIIpan-4.0 are the currently recommended algorithms in Immune Epitope Database (IEDB). They demonstrate cutting-edge performance and surpass other algorithms in benchmark experiments (Reynisson et al., 2020).

For predicting the linear B-cell epitopes, the IEDB with the method of Bepipred linear epitope prediction tool (v2.0) was utilized (Jespersen et al., 2017). The BepiPred-2.0 server performs the prediction of B-cell epitopes from a protein sequence *via* a random forest algorithm trained on epitopes and non-epitope amino acids determined from crystal structures. The threshold was set at 0.6 meaning that the residues scoring above the threshold were predicted to be part of the epitopes. The B-cell epitopes were then selected.

### 2.3. Multi-epitope DNA vaccine design

After the prediction of B-cell, MHC-I, and MHC-II epitopes, the epitopes with the most optimal features were selected. Multi-peptide vaccine construct was designed and epitopes of A5L, A15L, A35R, and B6R were fused *via* the appropriate linkers. The linkers play a crucial role in multi-epitope vaccine development as their reasonable selection allows to enhance structural stability, flexibility, and proper folding, as well as to increase the immune response by allowing to include multiple epitopes in a single vaccine construct. Thus, the well-described linkers KK, GPGPG, and EAAAK were used to connect different peptide components. EAAAK is a rigid linker that provides an alpha helix-forming structure between the domains that enhances the stability and maintains the constant spacing between the domains of the fusion protein as well as preserves their functions (Chen et al., 2013). B-cell epitopes were linked with a flexible KK linker (Rahmani et al., 2021; Tarrahimofrad et al., 2021). The KK linker allows the presentation of each linked epitope to antibodies while avoiding antibody induction against the whole joined sequences resulting in the antibody reactivity to each of the B-cell epitopes (Yano et al., 2005). MHC-I and MHC-II epitopes were conjugated together with another flexible linker GPGPG which is a glycine-rich linker, in addition enhancing the solubility of vaccine construct, and also provides high accessibility and flexibility for the adjacent epitopes (Dong et al., 2020; Khan et al., 2021; Martinelli, 2022). Except for the epitopes, to rationally optimize the fused multi-peptide construct for the optimal immunogenic outcome, adjuvants were also fused. Particularly, 45 amino acid long human β-defensin 3 (hBD3) with immunomodulatory effect (Hoover et al., 2003; Ali et al., 2017; Qamar et al., 2020) was adjoined to the N-terminal of the vaccine after the leading sequence of tissue plasminogen activator which also facilitates the antigen presentation (Ahammad and Lira, 2020). hBD3 is one of the top five adjuvants utilized for COVID-19 subunit vaccines (Mekonnen et al., 2022). Furthermore, defensins play a crucial role in defending against pathogen infections as they effectively bridge the innate and adaptive immune responses through leukocytes such as dendritic cells (DCs) and T cells (Bellamkonda et al., 2022). The rigid linker EAAAK (Nezafat et al., 2014; Tarrahimofrad et al., 2021) was used to conjugate the hBD3 with the pan-HLA-DR-binding epitope (PADRE) which triggers antigen-specific CD4+ T cells (Alexander et al., 2000). EAAAK was used to connect the PADRE sequence to the rest of the construct as well. For future *in vitro* validation of the expression of the multi-epitope construct, the C-terminal polyhistidine tag-−6xHis—was also adjoined (Boyoglu-Barnum et al., 2021). The untranslated regions (UTRs) were included for ensuring the proper stability and translation of the multi-epitope construct when it is transcribed into mRNA and for allowing the translation efficiency and stability of mRNA while validating the protein expression *in vitro* and *in vivo*. The following UTRs were used: cytomegalovirus (HCMV) immediate early (IE) partial sequence as 5′UTR, and human growth hormone partial sequence as a 3′UTR as they have demonstrated the effective stimulation of the protein production *in vivo* (Rybakova et al., 2019).

### 2.4. Vaccine properties—*In silico* assessment

The vaccine should have wide human population coverage. Thus, the IEDB population coverage analysis tool was employed to evaluate the worldwide human population coverage of the designed DNA vaccine (Bui et al., 2006). The allergenicity of the designed vaccine was predicted *via* the AllerCatPro server which predicts the allergenic proteins based on the similarity of both their amino acid sequences and 3D structures compared with the comprehensive dataset of already known allergens from the WHO/International Union of Immunological Societies, Comprehensive Protein Allergen Resource, Food Allergy Research and Resource Program, UniProtKB, and Allergome (Maurer-Stroh et al., 2019; Nguyen et al., 2022). VaxiJen v2.0 was applied to predict the antigenicity of the multi-epitope protein construct (Doytchinova and Flower, 2007a,b). The threshold was set as 0.4, and default parameters were employed (Ghafouri et al., 2022).

The multi-epitope vaccine physico-chemical properties were assessed by ProtParam (Gasteiger et al., 2005). Molecular weight (MW), the composition of atoms and amino acids, instability, estimated half-life, theoretical isoelectric point (pI), aliphatic indexes, and grand average of hydropathicity (GRAVY) of the vaccine construct were evaluated. The MW was determined by summing amino acids' average isotopic masses and the average isotopic mass of a single water molecule. pI was calculated by amino acids' pKa value. Half-life estimation describes the necessary time from protein synthesis until the disappearance of its half-amount from the cell. The instability index was used to determine how stable is the protein in a test tube. The value <40 estimates the protein as stable. GRAVY calculates the hydropathicity index of all amino acids divided by the length of the sequence. The larger number denotes more hydrophobicity of amino acids (Gasteiger et al., 2005). An aliphatic index is used to characterize the relative volume of protein which is occupied by aliphatic side chains and is considered a positive factor in increasing thermostability. The solubility of the vaccine protein was predicted *via* DeepSoluE. This server is a deep-learning predictor, and its prediction algorithm outperforms the other servers for the prediction of protein solubility (Wang and Zou, 2023).

### 2.5. Prediction and quality assessment of the vaccine's 3D structure

The 3D structure of the designed vaccine was constructed *via* RoseTTAFold (Baek et al., 2021). This tool uses a three-track network: integrating protein sequence patterns, amino acid interactions, and 3D structure to accurately predict protein structure and interaction (Baek et al., 2021). After the 3D structure of the vaccine protein was predicted, it was optimized with GalaxyRefine and FG-MD which is a molecular dynamics (MD)-based algorithm for protein structure refinement at the atomic level (Zhang et al., 2011; Heo et al., 2013). This immunoinformatics approach uses molecular dynamics simulation and relaxes and refines the protein structure (Heo et al., 2013). Predicting and knowing the potential structure is important as it determines the function of the protein that allows its application. As the predicted 3D structure provides the understanding of its interactions with the immune system, it is then used for further analyses, e.g., prediction of B-cell discontinuous epitopes and molecular docking. The tertiary structure quality of the 3D protein was then verified with the Ramachandran plot and ERRAT. The Ramachandran plot illustrates energetically permissible regions for the backbone dihedral angles ψ and ϕ of amino acid residues within a protein structure allowing the assessment of 3D protein structure quality (Nelson et al., 2021). ERRAT also represents the quality but for non-bonded interactions, and a higher score of ERRAT indicates a higher quality of the tertiary structure of the protein.

### 2.6. Presence of conformational B-cell epitopes in the vaccine

To predict the discontinuous B-cell epitopes, ElliPro was employed. This tool is the recourse of the immune epitope database (IEDB). It allows for predicting the conformational B-cell epitopes according to the antigen's tertiary structure. ElliPro associates predicted discontinuous epitopes with protrusion index (PI) which is averaged over epitopes' residues. The approximation of the antigen's tertiary structure is achieved *via* the number of ellipsoids. The PI score is defined based on the residue's mass center which is lying outside the largest ellipsoid. A higher value suggests higher solvent accessibility which, on the other hand, is crucial in protein stability and folding (Ponomarenko et al., 2008).

### 2.7. *In silico* immunization

The C-ImmSim server was utilized to analyze the simulated humoral and cellular immune responses elicited against the multi-epitope vaccine protein. After submitting the sequence of vaccines in the FASTA format, the server predicts the immune responses *via* a position-specific scoring matrix and machine learning algorithms (Rapin et al., 2011). Two HLA-A (01:01 and 02:01), two HLA-B (07:02 and 08:01), and two DRB (07:01 and 15:01) were selected through Allele Frequency Net Database (AFND). The prime, second, and booster doses of the vaccine were injected with 4 weeks gap. The volume and steps of the simulation were 10 and 270, respectively. The vaccine did not contain LPS. The random seed was 12,345, and the injection time periods were 1, 85, and 169 (Rapin et al., 2010).

### 2.8. Docking of vaccine antigen with host receptors

To evaluate the binding capacity of the vaccine antigen to its recognition receptors of the host, molecular docking analysis was conducted by the server of ClusPro. ClusPro determines the molecular docking of two proteins providing an outcome *via* presenting putative protein complexes in a ranked list. Ligand conformation, orientation, and position along with the assessment of binding affinity are the major properties that determine molecular docking. Ultimately, the electrostatic-favored protein–protein docked complexes with favorable desolvation-free energies are picked (Kozakov et al., 2017). Analysis of vaccine antigen's docking with MHC-I (HLA-A^*^02:01) (6TDS) and MHC-II (HLA-DRB1^*^01:01) (1AQD) host receptors was performed. The MHC molecules were used to dock the following epitopes in the vaccine construct: MHC-I ligands—ETNDLVTNVY (A5L), SPTRTWKVL (A15L), DSDVSQEVRKY (A35R), and VSDYVSELY (B6R); MHC-II ligands—APILLPSSTAPVLKP (A5L) and HSDYKSFEDAKANCA (A35R). The tertiary structure of these epitopes was predicted with AlphaFold2 (Jumper et al., 2021; Mirdita et al., 2022). The protein databank (PDB) files 6TDS (MHC-I) and 1AQD (MHC-II) were edited and cleaned to remove heteroatoms, bound peptides, and water molecules. The server of ClusPro performs the docking of rigid body *via* sampling billions of conformations, clusters 1000 lowest energy structures to find the most favorable models, and refines selected structures *via* minimization of energy (Kozakov et al., 2017).

## 3. Results

### 3.1. Design of the final construct

**Figure 1:**
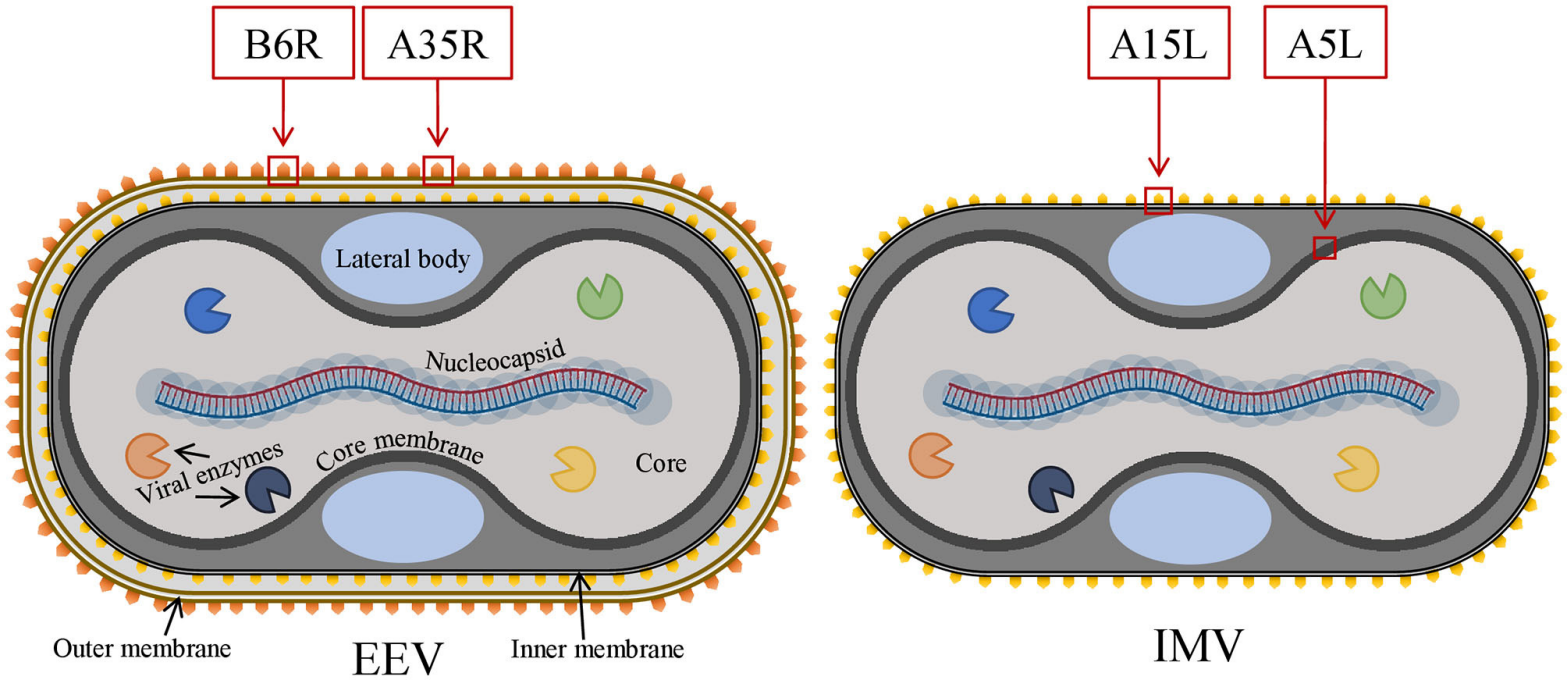
The general structure of two infectious forms of the mpox virus and selected viral antigens for vaccine design. Mpox, monkeypox.

**Figure 2:**
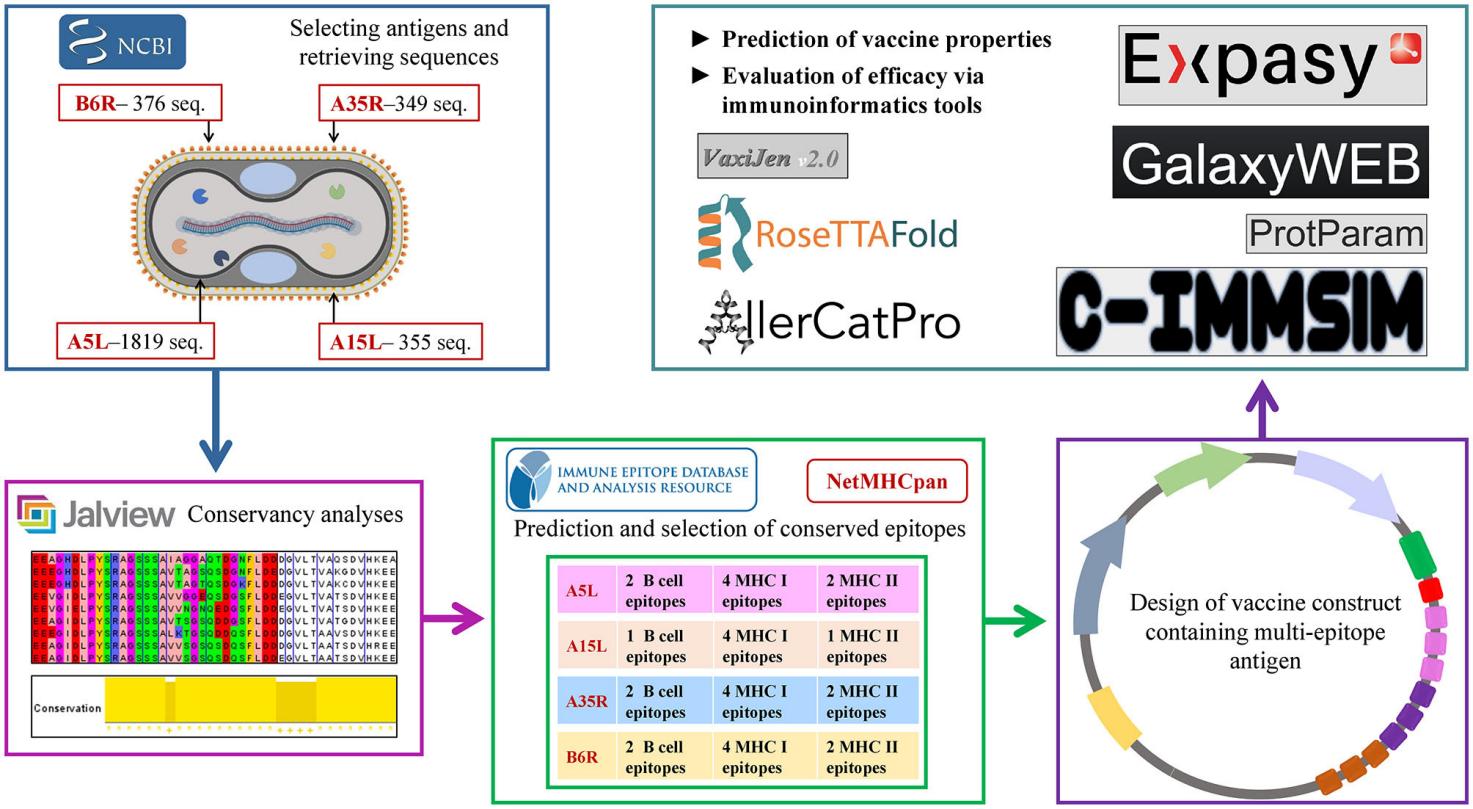
Flow diagram of DNA vaccine design strategy.

**Figure 3:**
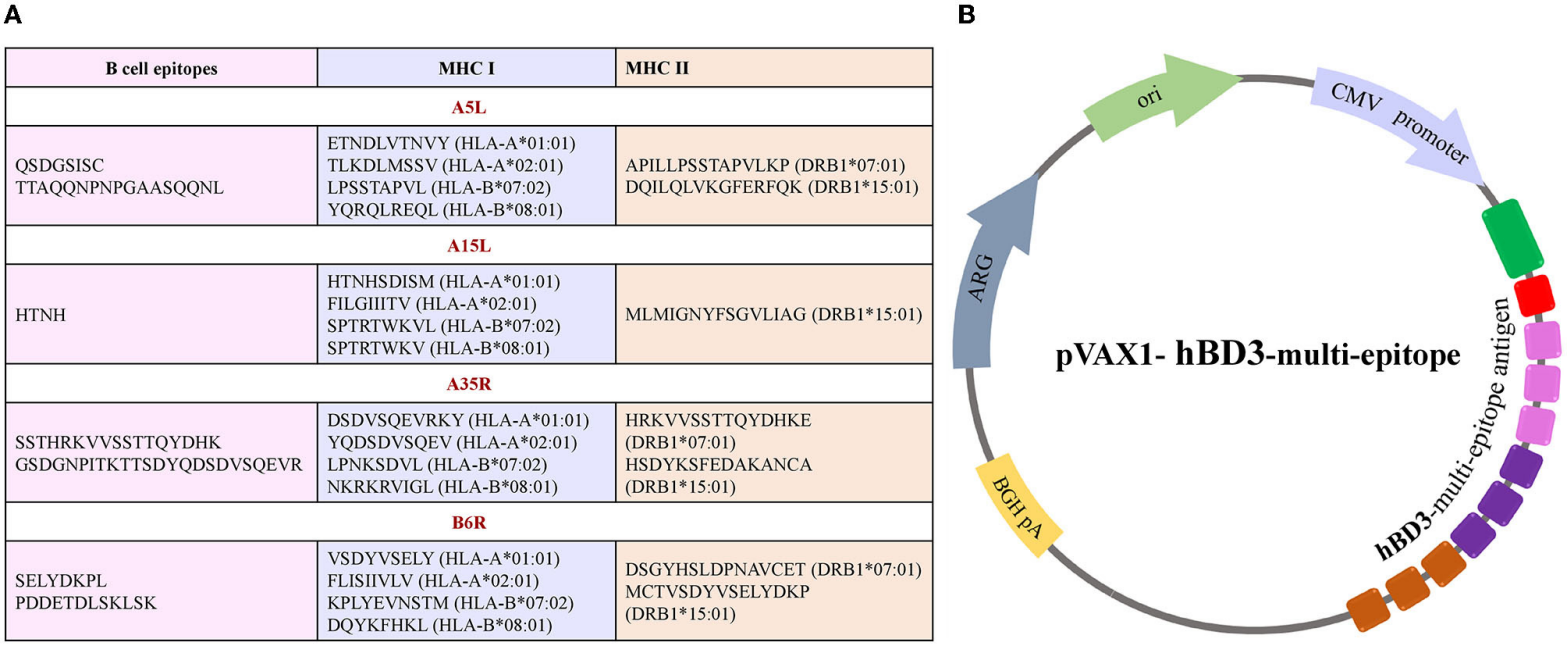
Illustration of multi-epitope DNA vaccine construct. **(A)** List of conserved epitopes of four selected antigens that were used for the vaccine design with appropriate MHC molecule alleles. **(B)** A scheme of plasmid DNA containing multi-epitope antigen of the mpox virus.

All the predicted B-cell, MHC-I, and MHC-II epitopes are given in Supplementary materials 1–12. In total, 30 epitopes were used for the final vaccine construct design. The open reading frame (ORF) of the vaccine construct is shown in Supplementary material. The selected four antigens of the mpox virus are shown in Figure 1. The location, functions, and immune properties of the selected antigens are listed in Table 1. The strategy of *in silico* design and evaluation of a potentially universal DNA vaccine for mpox are shown in Figure 2. All the epitopes included in the proposed multi-epitope vaccine construct along with the DNA vaccine are given in Figure 3.

**Table 1:** Selected antigens of the mpox virus and their functions.

**Antigen name**	**Location**	**Function/properties**	**Ref**.
A5L	IMV	Immunodominant virion core protein (281 aa) needed for the assembly and disassembly of virion; A highly antigenic protein of the viral core; Enhances the effect of cytotoxic T lymphocytes in the mpox challenge model; Similar to VACV A4L protein.	(Maa and Esteban, 1987; Shchelkunov et al., 2002; Hirao et al., 2011)
A15L	IMV	Essential inner membrane protein (90 aa) of IMV; Immunodominant antigen in smallpox vaccine; Similar to VACV A14L protein.	(Shchelkunov et al., 2002; Meng et al., 2018)
A35R	EEV	Envelope glycoprotein (181 aa); Cell-to-cell spread of virion; Neutralizing antibody target; Similar to VACV A33R protein	(Shchelkunov et al., 2002; Hirao et al., 2011)
B6R	EEV	Palmitoylated glycoprotein (317 aa); Required for efficient cell spread; Complement control; Similar to VACV B5R protein	(Shchelkunov et al., 2002; Hirao et al., 2011)

Mpox, monkeypox; IMV, intracellular mature virus; EEV, extracellular enveloped virus.

The population coverage analyses demonstrated that the 5 MHC-I alleles (HLA-A^*^01:01, HLA-A^*^02:01, HLA-B^*^07:02, HLA-B^*^08:01, and HLA-A^*^02:12) with IC50 binding affinity <500 nM with the MHC-I epitopes and the 13 MHC-II alleles (HLA-DRB1^*^07:01, HLA-DRB1^*^01:01, HLA-DRB1^*^09:01, HLA-DRB1^*^10:01, HLA-DRB1^*^04:01, HLA-DRB1^*^15:01, HLA-DRB1^*^14:01, HLA-DRB1^*^13:01, HLA-DRB1^*^08:01, HLA-DRB1^*^03:01, HLA-DRB1^*^11:01, HLA-DRB1^*^12:01, and HLA-DRB1^*^16:01) with IC50 binding affinity <500 nM with MHC-II epitopes cover 95.21% of the human population, which means that these epitopes could cover approximately 95% of the population from different regions of the world (Supplementary Figure 1 and Supplementary Table 1).

### 3.2. Analyses of protein structure and assessment of physico-chemical characteristics

**Figure 4:**
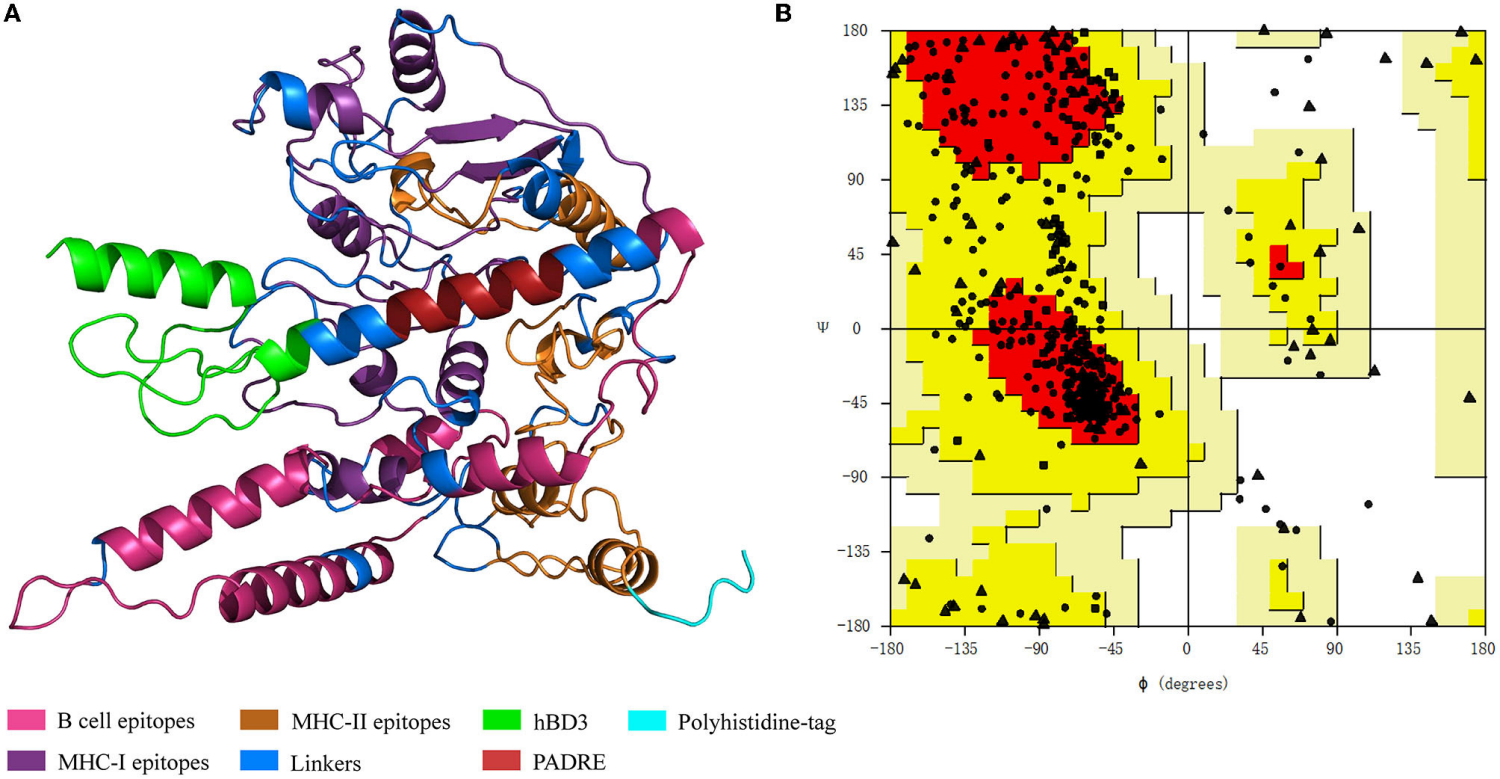
Vaccine protein structure and Ramachandran plot. **(A)** Vaccine protein tertiary structure. hBD3, human β-defensin 3; PADRE, pan-HLA-DR-binding epitope. **(B)** Ramachandran plot for the vaccine structure quality evaluation. The dots indicate amino acids. Dots' locations denote amino acids' backbone dihedral angles ψ against ϕ.

The predicted 3D structure of the vaccine construct along with the quality evaluation is given in Figure 4. The distribution of amino acid residues in the Ramachandran plot was as follows: refined structure had 279 residues in the most favored region, 93 residues in the additionally allowed region, 13 in the generously allowed region, and only 7 residues in the disallowed region. The Ramachandran plot allows us to visualize the energetically allowed and disallowed regions for the dihedral angles. For example, in poor quality homology models, many dihedral angles can be found in the forbidden regions of the plot which indicates the problems with the structure. The favored regions in the Ramachandran plot correspond to the regular secondary structures. The ERRAT score was 88.7218 meaning that all these parameters indicate the favorable overall quality of the vaccine antigen protein.

The instability index of the proposed vaccine construct was 29.12, which classifies the vaccine as stable. The aliphatic index was 60.22 which indicates the high thermostability of the vaccine protein. GRAVY was −0.678, stipulating hydrophilicity. The half-life *in vitro* was estimated to be 30 h, while the *in vivo* in the yeast and *E. coli* were estimated to be over 20 h and over 10 h, respectively. The number of amino acids was 542, MW was 56.55076 kDa, and the theoretical pI was 9.28. The vaccine construct contains 46 negatively charged amino acid residues (Asp+Glu) and 64 positively charged amino acid residues (Arg+Lys). The probability of the vaccine solubility was 0.7963, indicating the solubility of the vaccine protein (Wang and Zou, 2023).

### 3.3. Presence of discontinuous B-cell epitopes

**Figure 5:**
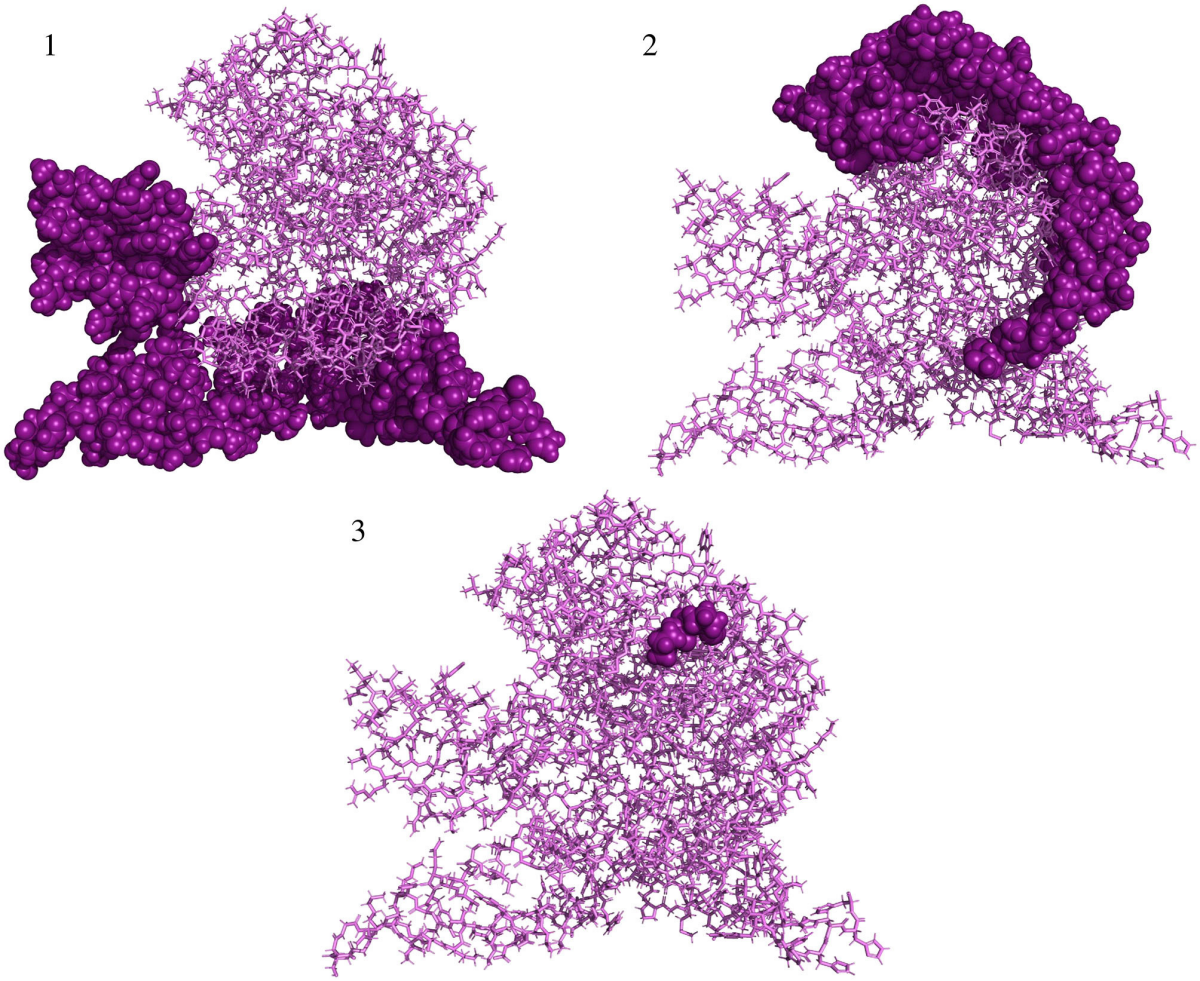
Predicted conformational B-cell epitopes localized in the structure of vaccine protein. Deep purple shows conformational epitopes, and violet denotes the tertiary structure of the vaccine protein.

All three conformational epitopes along with the scores are listed in Table 2. The locations of each discontinuous epitope in the tertiary structure of the antigen protein are illustrated in Figure 5.

**Table 2:** Predicted discontinuous B-cell epitopes.

**#**	**Epitopes**	**Residue number**	**Score**
1	A:G1, A:I2, A:I3, A:N4, A:T5, A:L6, A:Q7, A:K8, A:Y9, A:Y10, A:C11, A:V13, A:R14, A:G16, A:R17, A:C18, A:A19, A:V20, A:L21, A:S22, A:C23, A:L24, A:E28, A:Q29, A:I30, A:G31, A:K32, A:C33, A:S34, A:T35, A:R36, A:G37, A:R38, A:K39, A:C40, A:C41, A:R42, A:K109, A:V110, A:V111, A:S112, A:S113, A:T114, A:T115, A:Q116, A:Y117, A:D118, A:H119, A:K120, A:K121, A:K122, A:G123, A:S124, A:D125, A:G126, A:N127, A:P128, A:I129, A:T130, A:K131, A:T132, A:T133, A:S134, A:D135, A:Y136, A:Q137, A:D138, A:S139, A:D140, A:V141, A:S142, A:Q143, A:E144, A:V145, A:R146, A:K147, A:K148, A:S149, A:E150, A:L151, A:Y152, A:D153, A:K154, A:P155, A:L156, A:K157, A:K158, A:P159, A:D160, A:D161, A:E162, A:T163, A:D164, A:L165, A:K167, A:L168, A:K170, A:G171, A:P172, A:G173, A:P174, A:G175, A:D179, A:P203, A:G204, A:Y472, A:H474, A:K475, A:E476, A:G477, A:Y485, A:S487, A:F488, A:G499, A:P500, A:G501, A:D502, A:S503, A:G504, A:Y505, A:H506, A:S507, A:L508, A:D509, A:P510, A:N511, A:A512, A:V513, A:C514, A:E515, A:T516, A:G517, A:P518, A:G519, A:P520, A:G521, A:M522, A:C523, A:T524, A:V525, A:S526, A:D527, A:Y528, A:V529, A:S530, A:E531, A:L532, A:Y533, A:D534, A:K535, A:P536, A:H537, A:H538, A:H539, A:H540, A:H541, A:H542	157	0.746
2	A:A67, A:K68, A:Q69, A:S70, A:D71, A:G72, A:S73, A:I74, A:S75, A:C76, A:K78, A:T79, A:T80, A:A81, A:Q82, A:Q83, A:N84, A:P85, A:N86, A:P87, A:G88, A:A89, A:Q92, A:G272, A:P273, A:G274, A:S275, A:P276, A:T277, A:R278, A:T279, A:W280, A:K281, A:V282, A:G283, A:P284, A:G285, A:P286, A:G287, A:D288, A:S289, A:D290, A:V291, A:S292, A:Q293, A:E294, A:V295, A:R296, A:K297, A:Y298, A:G299, A:P300, A:G301, A:P302, A:G303, A:Y304, A:Q305, A:D306, A:S307, A:D308, A:V309, A:S310, A:Q311, A:E312, A:V313, A:G314, A:P315, A:G316, A:P317, A:G318, A:L319, A:P320, A:N321, A:K322, A:S323, A:D324, A:V325, A:L326, A:G327, A:P328, A:G329, A:P330, A:G331, A:N332, A:K333, A:R334, A:K335, A:R336, A:I338, A:G339, A:L340, A:G341, A:Y354, A:G355, A:P356, A:G357, A:V368, A:G369, A:P370, A:G371, A:P372, A:G373, A:K374, A:P375, A:G384, A:P385, A:G386, A:P387, A:G388, A:H394, A:K395, A:L396, A:G397, A:P398, A:G399, A:P400, A:G401, A:P403, A:D422, A:K429, A:E432, A:R433, A:Q435, A:K436, A:G437, A:P438, A:G439, A:P440, A:G446, A:G456, A:G457, A:P458, A:G459, A:P460	134	0.663
3	A:P416, A:G417, A:P418	3	0.504

### 3.4. Assessment of allergenicity, antigenicity, and molecular docking

A vaccine protein construct did not show evidence of allergenicity allowing us to presume that the multi-epitope construct can be considered with low allergenic potential. On the other hand, the multi-peptide was demonstrated to be a probable antigen with a score of 0.4248.

**Figure 6:**
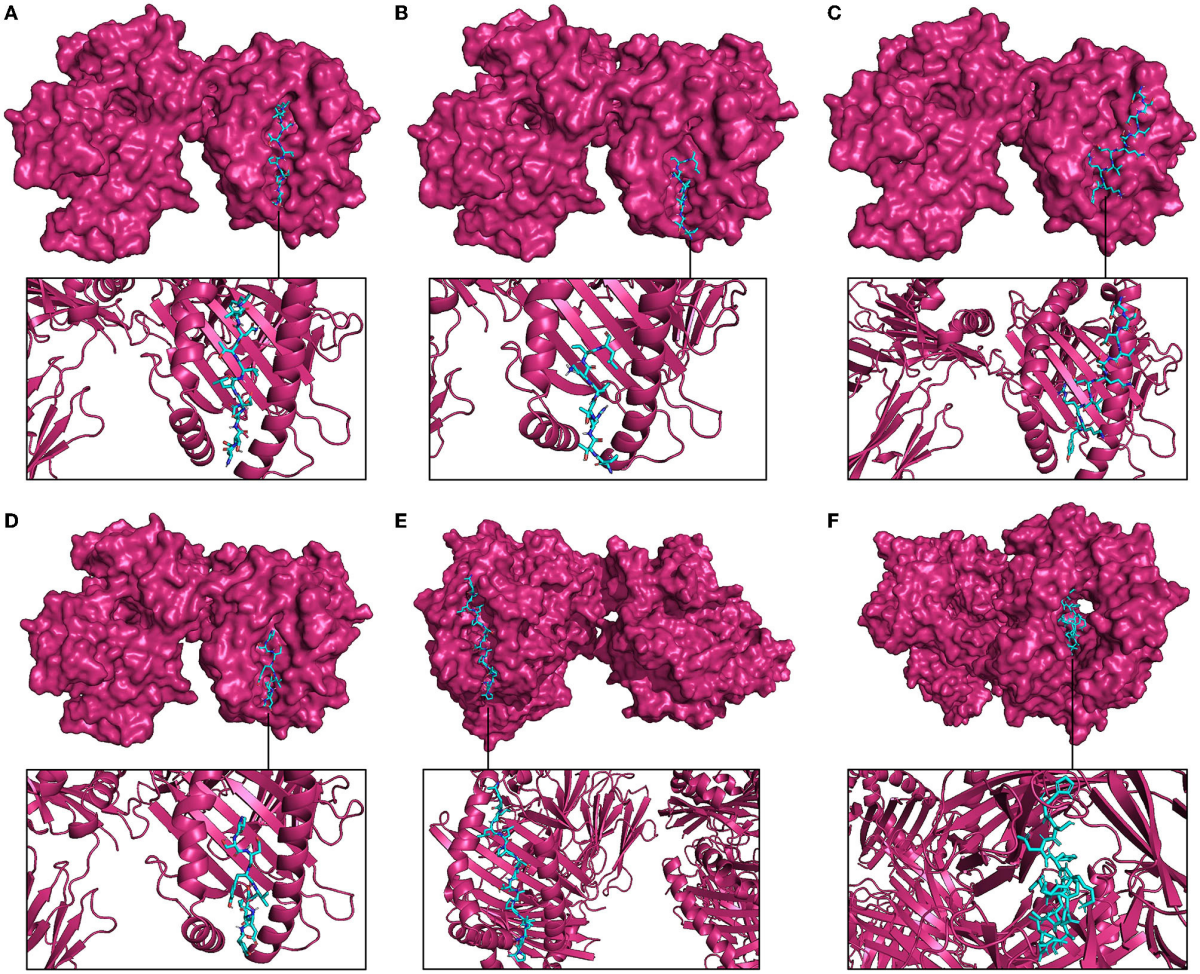
Molecular docking between representative epitopes (cyan) of vaccine construct and MHC molecules (magenta). **(A)** Docked complex of epitope ETNDLVTNVY (A5L) and MHC-I molecule. **(B)** Docked complex of epitope SPTRTWKVL (A15L) and MHC-I molecule. **(C)** Docked complex of epitope DSDVSQEVRKY (A35R) and MHC-I molecule. **(D)** Docked complex of epitope VSDYVSELY (B6R) and MHC-I molecule. **(E)** Docked complex of epitope APILLPSSTAPVLKP (A5L) and MHC-II molecule. **(F)** Docked complex of epitope HSDYKSFEDAKANCA (A35R) and MHC-II molecule.

The molecular docking with MHC molecules demonstrated stable binding. ClusPro identifies the most probable complex models by determining the largest clusters available. The interaction positions of the docked complex of vaccine epitopes and MHC molecules are shown in Figure 6. The interaction energy is given in Table 3 where the cluster size is the size of the largest cluster found, while the lowest energy is the Gibbs free energy of the best complex conformation (Kozakov et al., 2017).

**Table 3:** Molecular docking of selected vaccine epitopes and MHC molecules.

**Peptide**	**Cluster size**	**Center weighted score**	**Lowest energy**
**Molecular docking of epitopes with 6TDS (MHC-I)**			
ETNDLVTNVY	252	−909.7	−1,089.0
SPTRTWKVL	396	−1,083.7	−1,239.3
DSDVSQEVRKY	217	−647.4	−794.9
VSDYVSELY	344	−1,194.4	−1,194.4
**Molecular docking of epitopes with 1AQD (MHC-II)**			
APILLPSSTAPVLKP	190	−1,296.4	−1,296.4
HSDYKSFEDAKANCA	244	−845.1	−1,023.0

### 3.5. Evaluation of immune response induced by immune simulation

**Figure 7:**
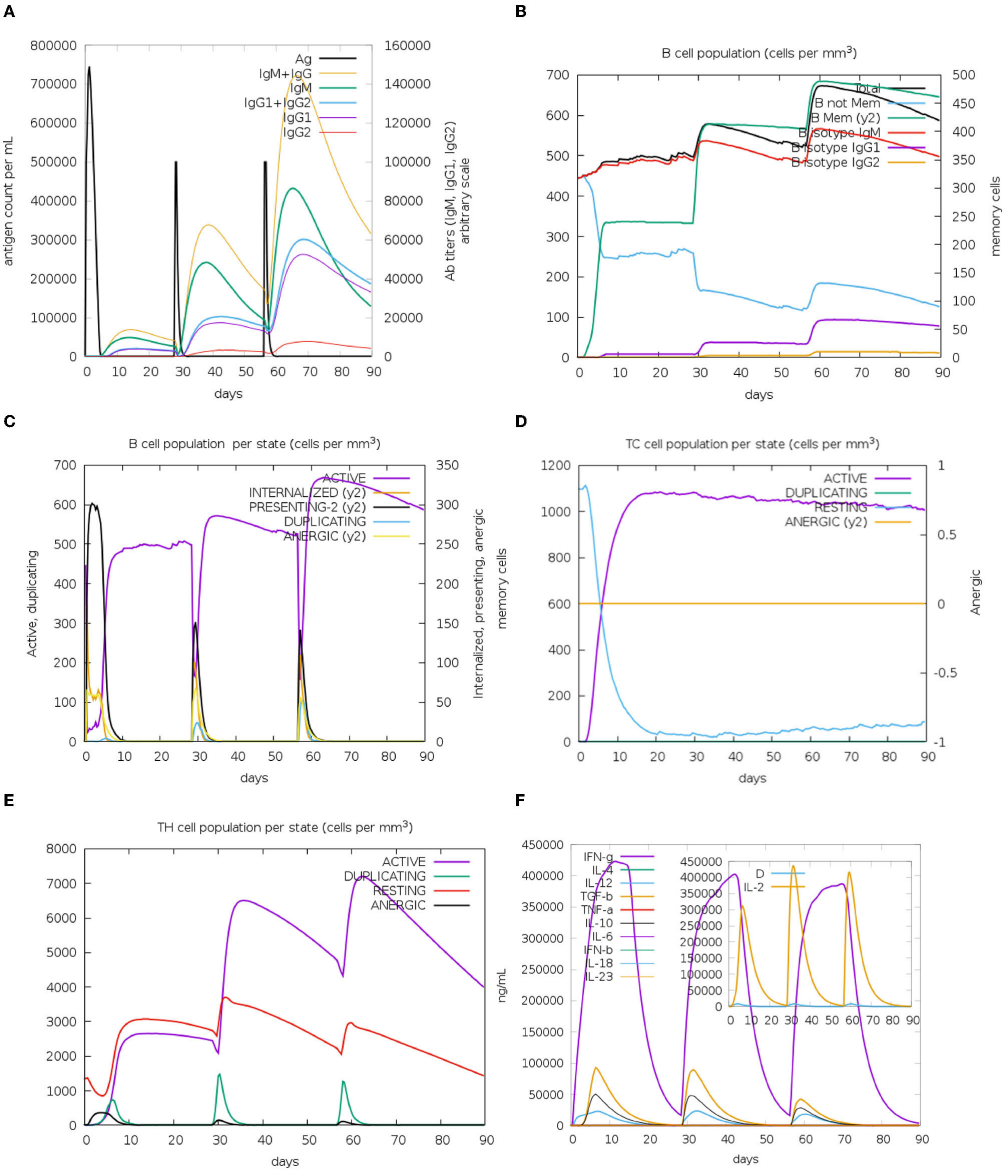
*In silico* immunization results. **(A)** Augmented antibody production. Immunoglobulin subclasses are indicated in different colors. **(B)** The population of B cells after three doses of vaccinations. **(C)** B-cell population per entity state. **(D)** Cytotoxic T (CT)-cell production in different states. The resting state shows CT cells not exposed to the antigen. Anergic CT cells' tolerance to the antigen because of repeated exposure. **(E)** TH cell production due to exposure to antigen. **(F)** Levels of cytokines upon three injections of the vaccine. The additional graph indicates the level of IL-2 and general danger signal—D, which represents an activator signal for macrophages.

*In silico* analyses demonstrated that the immune response triggered by the proposed vaccine was compatible with the responses generally induced *via in vivo* immunization. The immune responses after the additional two booster doses of vaccine were stronger compared to the prime immunization. The immunization lowered the antigen level, while high levels of antibodies (IgG and IgM) were produced. Immunoglobulin and antigen levels varied over time. The antigen abundance peaked at each injection time point (Figure 7A). The humoral response was induced after each shot, and the antibody levels remained elevated during the weeks after the last vaccine injection. The immunization stimulated the B cells (Figures 7B, C). The ignorable number of anergic cells and activation of CTLs was observed (Figure 7D). The counts of HTLs demonstrate that the duplication phase starts immediately after each injection (Figure 7E). Days after each injection and upon successful interaction with antigen-presenting cells, HTLs start to duplicate and differentiate into memory cells. HTLs also foster the secretion of cytokines and humoral response. High levels of IFN-γ, TGF-β, and IL-10 were induced upon each injection (Figure 7F). The other parameters of immune responses are shown in Supplementary Figure 2.

## 4. Discussion

Since the mpox virus crossed African borders and caused a multi-country outbreak (Supplementary Figure 3) with increased cases of human-to-human transmission, the global concern has increased; thus, there is a strong need for the development of specific drugs and vaccines. Although the available smallpox vaccine is effective for mpox prevention (Shafaati and Zandi, 2023), except for the painful immunization procedure, adverse effects were also reported (Maurer et al., 2003). Certain groups of the population are vulnerable to vaccination as serious side effects including pericarditis and myocarditis have been noted. A risk of recombination of genes between the mpox virus and attenuated or live poxvirus-based vaccines exists (Lum et al., 2022). In addition, after immunization with the smallpox vaccine, a manifestation of mpox disease still has been observed (Meyer et al., 2002). All the abovementioned disadvantages of the smallpox vaccine can be potentially overcome by the new DNA-based universal mpox vaccine candidate as, on the one hand, DNA vaccines are safe and simple to manufacture compared to their conventional counterparts. On the other hand, the mpox vaccine designed in this study has the potential to be effective against various strains of mpox viruses and smallpox virus since these two species share a high level of similarity. Noteworthily, the majority of the cases occur in men who have sex with men (MSM). Close physical contact is indeed crucial in transmission (Heskin et al., 2022; Martínez et al., 2022; Orviz et al., 2022). The sex-related infection tendency was always reported before the current multi-country outbreak (Sklenovská and Van Ranst, 2018).

The report of human-to-dog transmission of the mpox virus also complicates combating the outbreak (Seang et al., 2022). Additionally, the re-emergence risk of smallpox (mortality rate 10–75%) (Javier et al., 2022) or risk of spreading to the Central African clade of the mpox virus, which is much deadlier compared with the currently spread West African clade (Bunge et al., 2022), is very real and has a huge potential to cause severe outcomes. The recent multi-country outbreak also demonstrated that the mpox virus which belongs to the West African clade can be life-threatening (Meyer et al., 2002). All the abovementioned conditions indicate the urgency of designing a new, specific next-generation mpox vaccine that will be effective, safe, and easy to develop.

In general, nucleic acid-based approaches find ways through various applications such as vaccines against bacterial (Wang et al., 2023) and viral (Hobernik and Bros, 2018) infections, protein replacement (Papukashvili et al., 2022a; Vavilis et al., 2023), and cancer treatment (Hobernik and Bros, 2018; Liu et al., 2022a). *In silico*-designed and evaluated multi-peptide vaccines are thermodynamically stable, effective, specific, and easily and inexpensively developed compared with conventional vaccines.

In this study, four antigens of the mpox virus that are the target of immunity were selected according to their functions and role in immune response (Table 1). A5L is an immunodominant core protein that is necessary for the assembly and disassembly of the virion (Maa and Esteban, 1987; Shchelkunov et al., 2002). A4L, which is the ortholog of the A5L protein in the vaccinia virus, has been used as a part of the DNA vaccine along with the orthologs of A35R and B6R, among the other antigens and induced strong immune response in non-human primates, providing protection from the mpox virus (Hirao et al., 2011). A15L (similar to A14L in the vaccinia virus) is an inner membrane immunodominant protein which is a target of antibodies (Shchelkunov et al., 2002; Meng et al., 2018). In addition to the aforementioned rationale for selecting these antigens, another reason was to make a vaccine by combining these four antigens to assess the immune response. This approach aimed to generate an additional vaccine candidate as having multiple vaccine candidates would greatly enhance the effectiveness of pandemic prevention efforts. After the conservancy analyses of each protein sequence downloaded from the NCBI database, B- and T-cell conserved epitopes were predicted to design a DNA vaccine. DNA vaccines are a type of nucleic acid vaccine with a number of advantages over conventional immunization strategies (Supplementary Figure 4).

**Figure 8:**
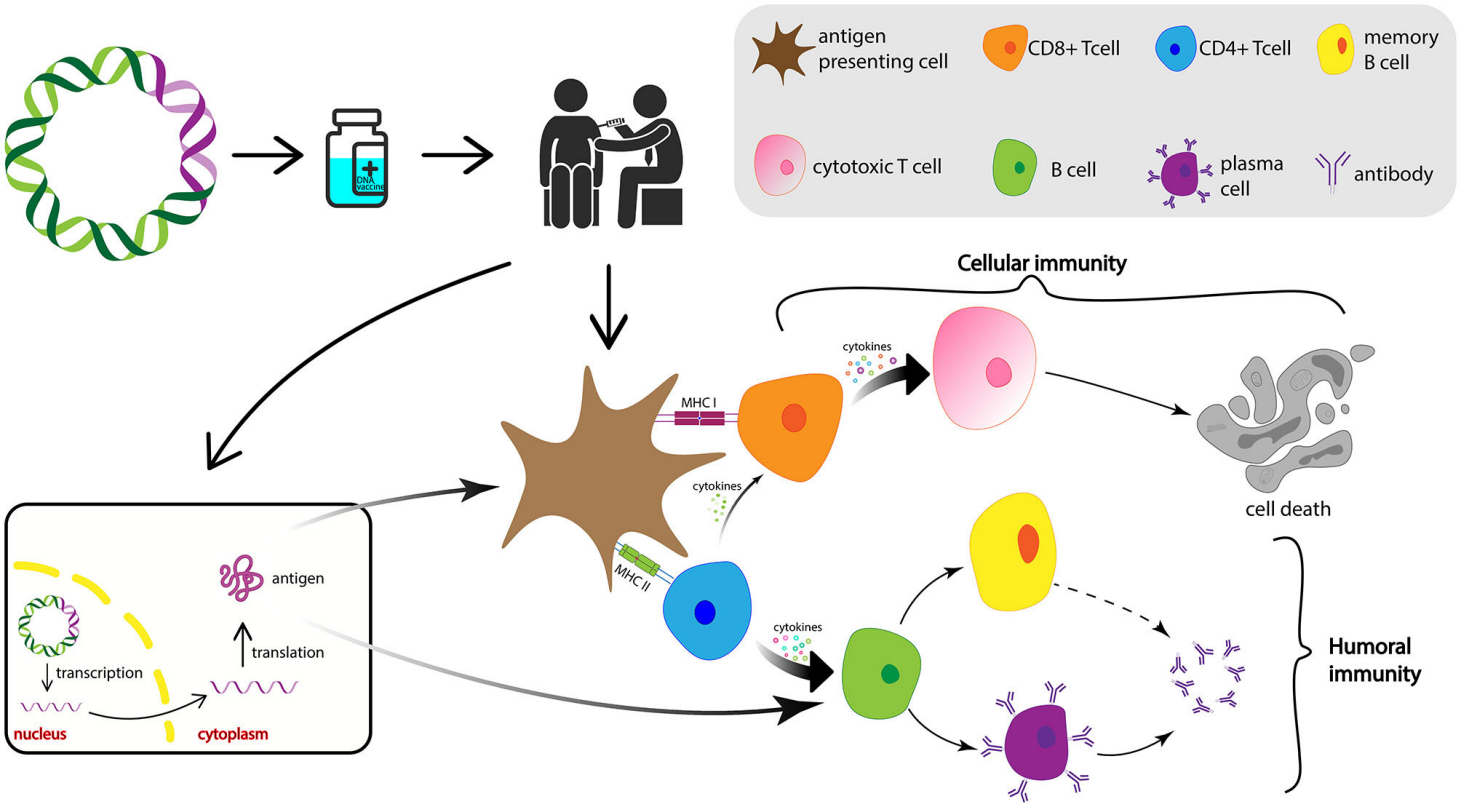
Diagrammatic representation of the mechanism of action of DNA vaccines. After the DNA vaccine is intramuscularly injected, it is delivered into the nucleus of the cells where its transcription takes place. After the mRNA transcribed from the vaccine DNA is transported into the cytoplasm, translation occurs and vaccine antigen is produced and released. Antigen then is recognized and phagocytosed by the APCs from where they can be processed into small peptides and presented on the cell surface *via* MHC-I or MHC-II, induce CTLs and HTLs, and activate cellular and humoral immune responses, respectively. APCs, antigen-presenting cells; MHC, major histocompatibility complex; CTL, cytotoxic T lymphocyte; HTL, helper T lymphocytes.

DNA vaccines instruct the host cells to produce antigens similar to the virus and the body becomes ready for the future possible infection, which is capable to stop the spreading of the virus and, thus, a manifestation of clinical symptoms (Chavda et al., 2021a; Qin et al., 2021). After the delivery into the host cell, the DNA is transported to the nucleus where transcription takes place. Subsequently, the mRNA is conveyed to the cytoplasm where it undergoes translation to produce the antigens for the vaccine. Figure 8 depicts the schematic representation of the mechanism of action for DNA vaccines upon delivery into the host cell.

**Figure 9:**
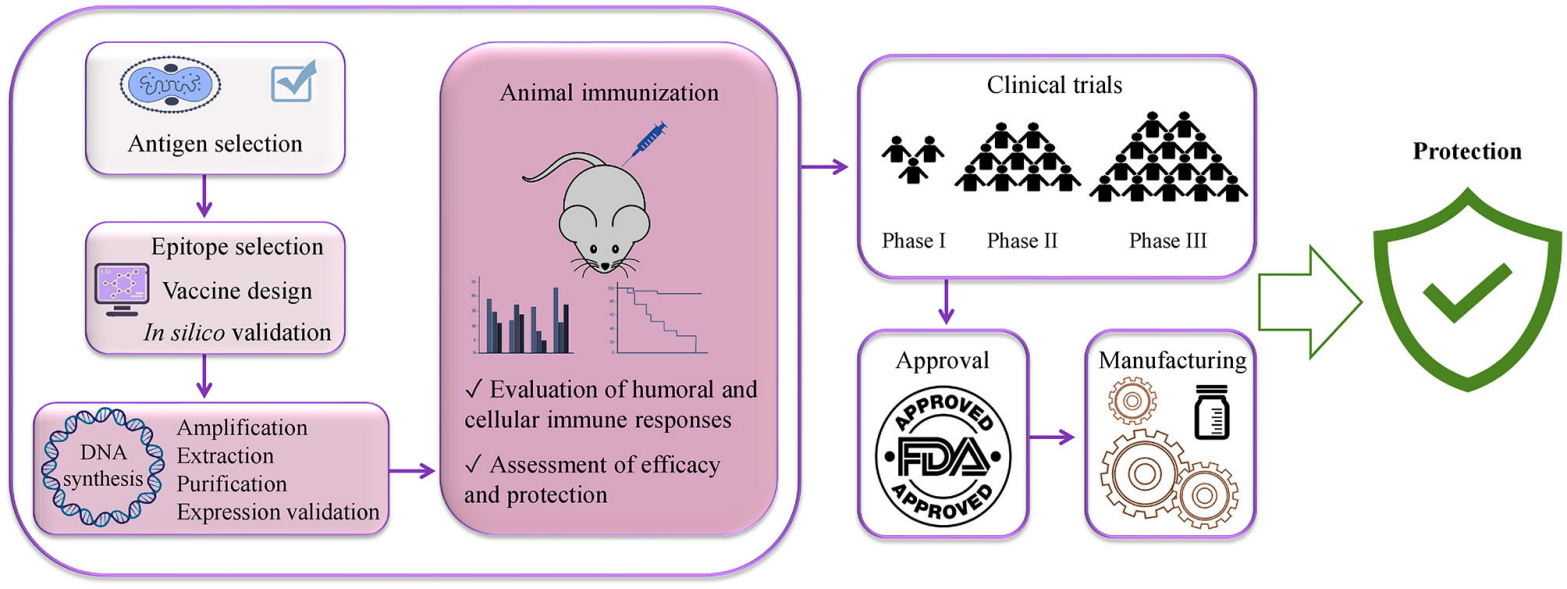
Steps needed for potentially universal DNA vaccine development using immunoinformatics approaches from vaccine antigen selection to the public availability of the vaccine.

Remarkably, although DNA vaccine enters the host nucleus, which is the main concern related to DNA vaccines, the likelihood of its integration into the host genome is extremely low (Williams, 2013). Indeed, there is a number of clinical trials on the application of DNA vaccines for the prevention of various infectious diseases: NCT04591184, NCT01498718, NCT01487876, and NCT04445389. Furthermore, India has designated the emergency use of the first DNA vaccine against COVID-19 in 2021 (Sheridan, 2021) and there are a number of other COVID-19 DNA vaccine candidates in clinical (Sheridan, 2021; Silveira et al., 2021) and preclinical development (Shafaati et al., 2022; Wang et al., 2022). More importantly, DNA vaccines have shown protective efficacy against the mpox virus in non-human primates (Hooper et al., 2004; Hirao et al., 2011). The steps of developing a DNA-based vaccine from the antigen selection to the commercial availability are vaccine design, optimization with proper linkers and addition of immunomodulator sequences, synthesis, transformation into competent bacterial cells for its amplification, extraction, purification, animal immunization, assessment of immune responses, clinical trials, approval, and manufacturing (Papukashvili et al., 2022b; Rcheulishvili et al., 2022). Steps that are needed for developing a potentially universal DNA-based anti-mpox multi-epitope vaccine are given in Figure 9.

Designing a vaccine employing immunoinformatics approaches significantly shortens the time of vaccine development and lays the groundwork for the rational design of the effective vaccine, while in the case of conventional techniques, the development of a vaccine may take decades of laboratory work (Rappuoli and Aderem, 2011). Leveraging immunoinformatics enables us to predict the potential outcome of the vaccine. Upon achieving favorable results with the designed vaccine, it becomes more rational to proceed with *in vitro* and *in vivo* screening of the multi-epitope construct.

Leveraging immunoinformatics tools enables us to forecast the potential efficacy of the vaccine. Upon achieving favorable outcomes with the designed vaccine, it becomes more logical to proceed with *in vitro* and *in vivo* screening of the multi-epitope construct.

The links of the employed servers and tools are given in Supplementary Table 2. Apparently, there are a number of *in silico* studies which designed the vaccine and computationally evaluated its efficacy, stability, etc., e.g., vaccines for COVID-19 (Dong et al., 2020; Oliveira et al., 2020), influenza (Behbahani et al., 2021; Sharma et al., 2021; Rcheulishvili et al., 2023), mpox (Hirao et al., 2011; Akhtar et al., 2022), and other viruses (Ali et al., 2019; Mahmudul et al., 2020; Ros-Lucas et al., 2020).

In this study, the vaccine construct designed here was found to have favorable physico-chemical properties and induce strong cellular and humoral immune responses. The immune simulation analysis shows that immunization with the multi-epitope vaccine candidate induces the production of HTLs and CTLs and stimulates the B-cell population, antibodies, and cytokines. The computationally designed vaccine in this study requires to be validated with *in vitro* and *in vivo* studies to confirm the outcomes obtained in this study.

## 5. Conclusion

In summary, the development of new, next-generation, specific vaccine candidates against the mpox virus is undoubtedly essential. The available immunoinformatics approaches allow for the rational design of the potentially protective vaccine and facilitate the process of vaccine development. The results obtained in this study demonstrate that the multi-epitope vaccine designed here may be suggested as an auspicious vaccine candidate which has the prospect of eliciting strong immune responses and providing protection against the mpox virus. In addition, the strategy of developing a universal multi-epitope DNA vaccine used in this study may have a positive impact on the development of a potentially universal vaccine against the mpox virus and other viruses and, thus, will aid in averting future outbreaks or pandemics.

## Data availability statement

The original contributions presented in the study are included in the article/Supplementary material, further inquiries can be directed to the corresponding authors.

## Author contributions

NR conceived and designed the study and wrote the original draft. NR and JM contributed to the methodology and investigation. NR, DP, and SF contributed to the visualization. CL and XW revised the manuscript. YH supervised the study. PW supervised and administered the project. All authors contributed to the article and approved the submitted version.
