# Supplementary material for: Design, evaluation, and immune simulation of potentially universal multi-epitope mpox vaccine candidate: focus on DNA vaccine
Source: Front Microbiol. 2023 Jul 21;14:1203355. doi: 10.3389/fmicb.2023.1203355 (PMC10403236; doi:10.3389/fmicb.2023.1203355)
Supplement: Supplementary file 13 [file Data_Sheet_1.docx]

Supplementary Material

Design, evaluation, and immune simulation of potentially universal multi-epitope mpox vaccine candidate: focus on DNA vaccine

Nino Rcheulishvili^†^, Jiawei Mao^†^, Dimitri Papukashvili, Shunping Feng, Cong Liu, Xingyun Wang, Yunjiao He*, Peng George Wang*

*** Correspondence:** Yunjiao He: heyj@sustech.edu.cn; Peng George Wang: wangp6@sustech.edu.cn

# Supplementary Data

# Open reading frame amino acid sequence of multi-epitope DNA vaccine construct

GIINTLQKYYCRVRGGRCAVLSCLPKEEQIGKCSTRGRKCCRRKKEAAAKAKFVAAWTLKAAAEAAAKQSDGSISCKKTTAQQNPNPGAASQQNLKKHTNHKKSSTHRKVVSSTTQYDHKKKGSDGNPITKTTSDYQDSDVSQEVRKKSELYDKPLKKPDDETDLSKLSKGPGPGETNDLVTNVYGPGPGTLKDLMSSVGPGPGLPSSTAPVLGPGPGYQRQLREQLGPGPGHTNHSDISMGPGPGFILGIIITVGPGPGSPTRTWKVLGPGPGSPTRTWKVGPGPGDSDVSQEVRKYGPGPGYQDSDVSQEVGPGPGLPNKSDVLGPGPGNKRKRVIGLGPGPGVSDYVSELYGPGPGFLISIIVLVGPGPGKPLYEVNSTMGPGPGDQYKFHKLGPGPGAPILLPSSTAPVLKPGPGPGDQILQLVKGFERFQKGPGPGMLMIGNYFSGVLIAGGPGPGHRKVVSSTTQYDHKEGPGPGHSDYKSFEDAKANCAGPGPGDSGYHSLDPNAVCETGPGPGMCTVSDYVSELYDKPHHHHHH

| Signal peptide of TPA | MDAMKRGLCCVLLLCGAVFVSPS |
| --- | --- |
| Human β-defensin 3 (hBD3) | GIINTLQKYYCRVRGGRCAVLSCLPKEEQIGKCSTRGRKCCRRKK |
| PADRE | AKFVAAWTLKAAA |

# Supplementary Figures and Tables

## Supplementary Figures


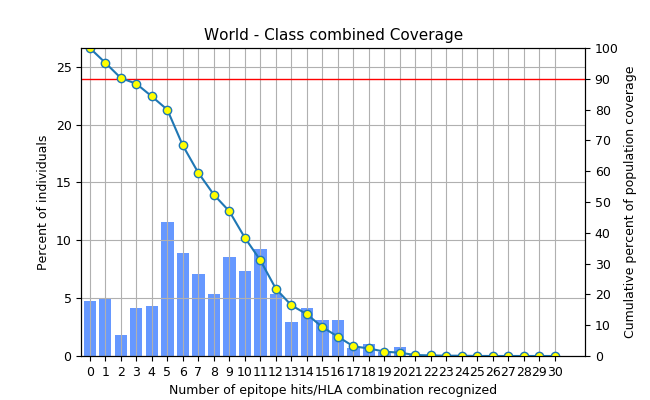


**Supplementary Figure 1.** Population Coverage Analysis. Prediction of the population coverage for epitopes with MHC-I and MHC-II alleles around the world.


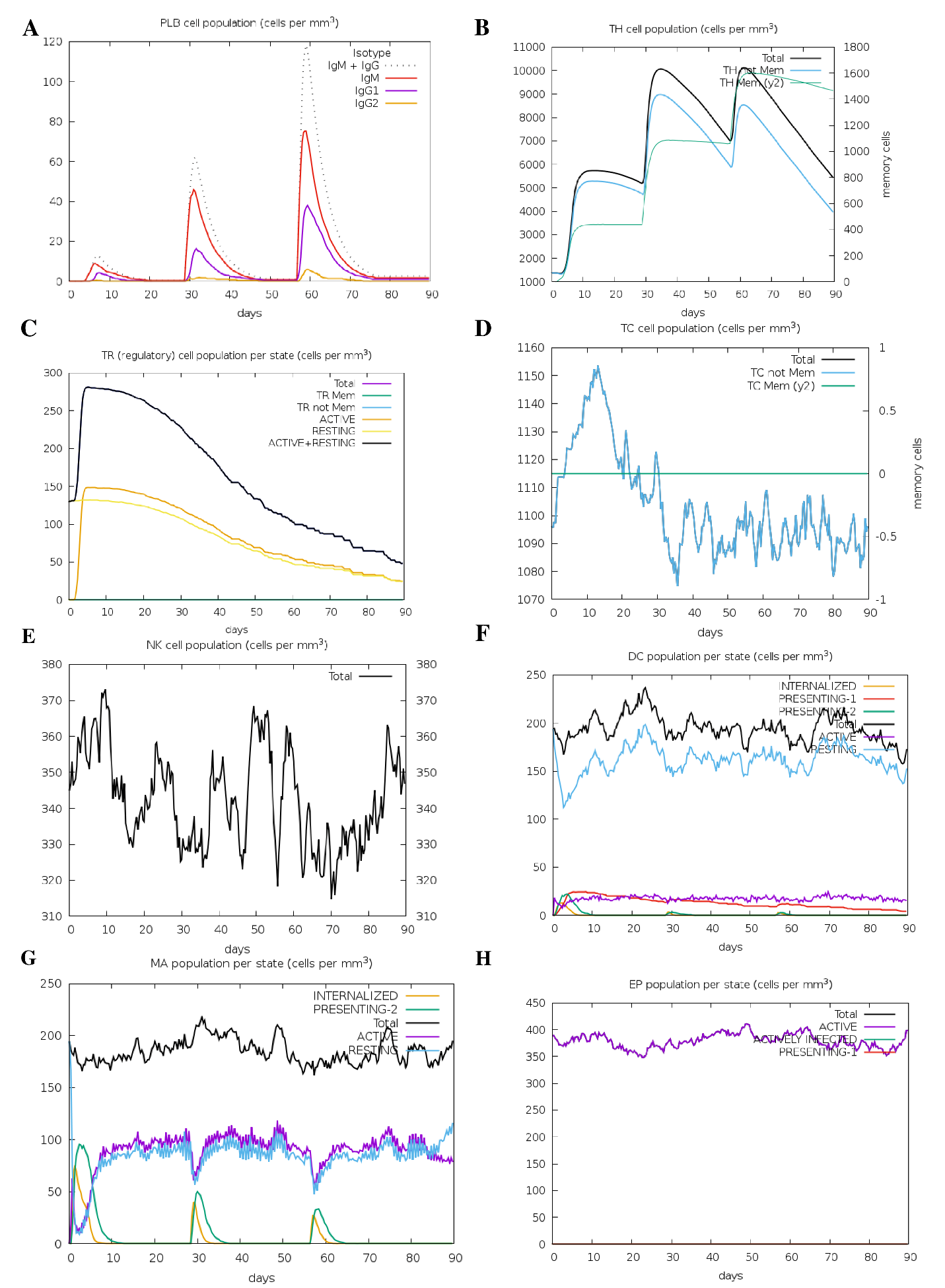


**Supplementary Figure 2.** Immune simulation results. (A) Plasma B lymphocytes count sub-divided per isotype (IgM, IgG1 and IgG2). (B) CD4 T-helper lymphocytes count. The plot shows total and memory counts. (C) Count of CD4 T-regulatory lymphocytes (total, memory, and per entity-state counts). (D) Count of CD8 T-cytotoxic lymphocytes. (E) Total count of Natural Killer cells. (F) Total count of active, resting, internalized, and antigen-presenting dendritic cells. (G) Total count of internalized, presenting on MHC class-II, active, and resting macrophages. (H) Total count broken down to active, virus-infected and presenting on MHC-I molecule epithelial cells.


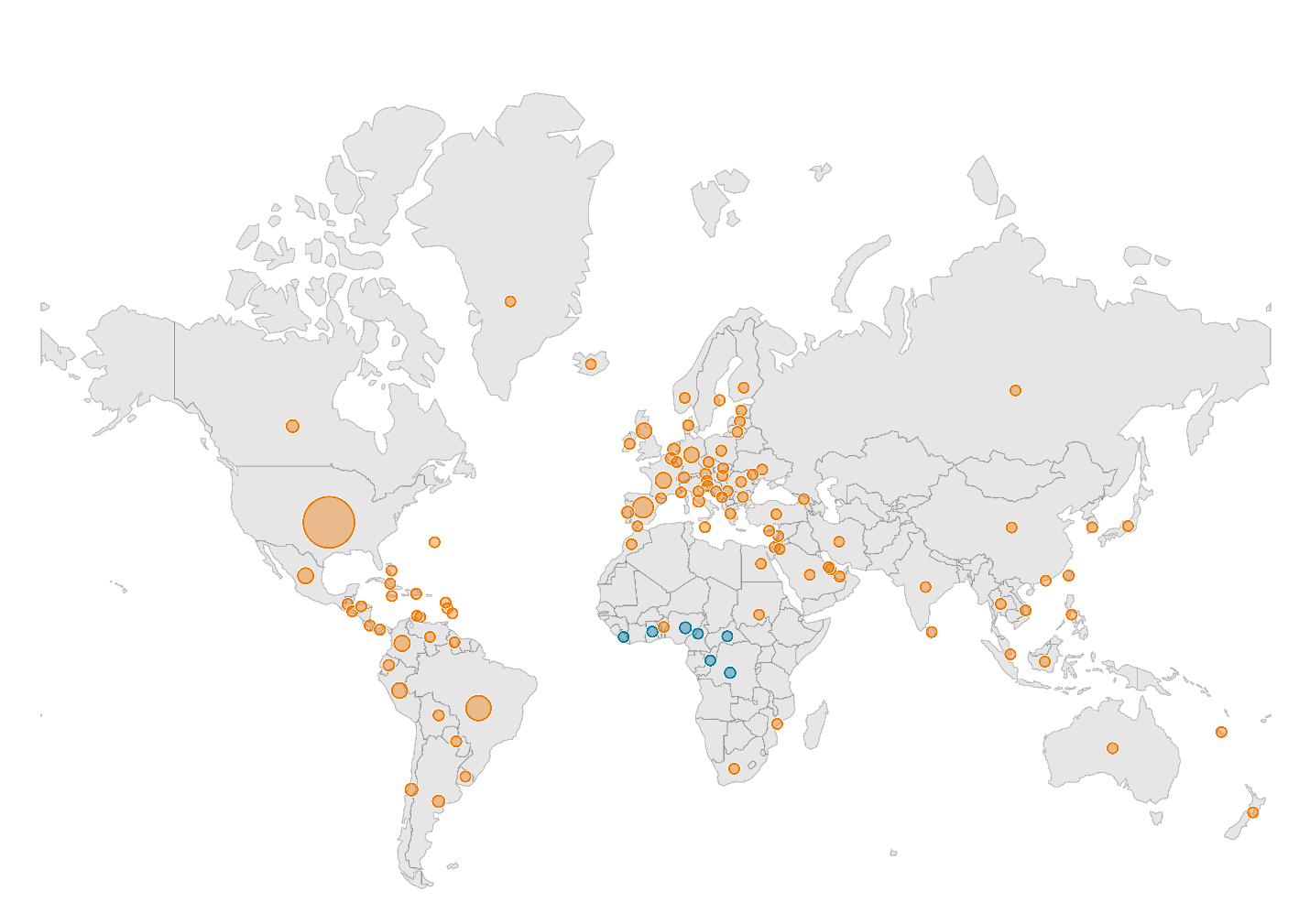


**Supplementary Figure 3.** Map of Mpox 2022 multi-country outbreak. Blue color indicates countries where mpox has been historically reported. Orange color indicates countries where mpox has not been reported historically.

Source: Centers for Disease Control and Prevention (CDC) https://www.cdc.gov/poxvirus/mpox/response/2022/world-map.html).


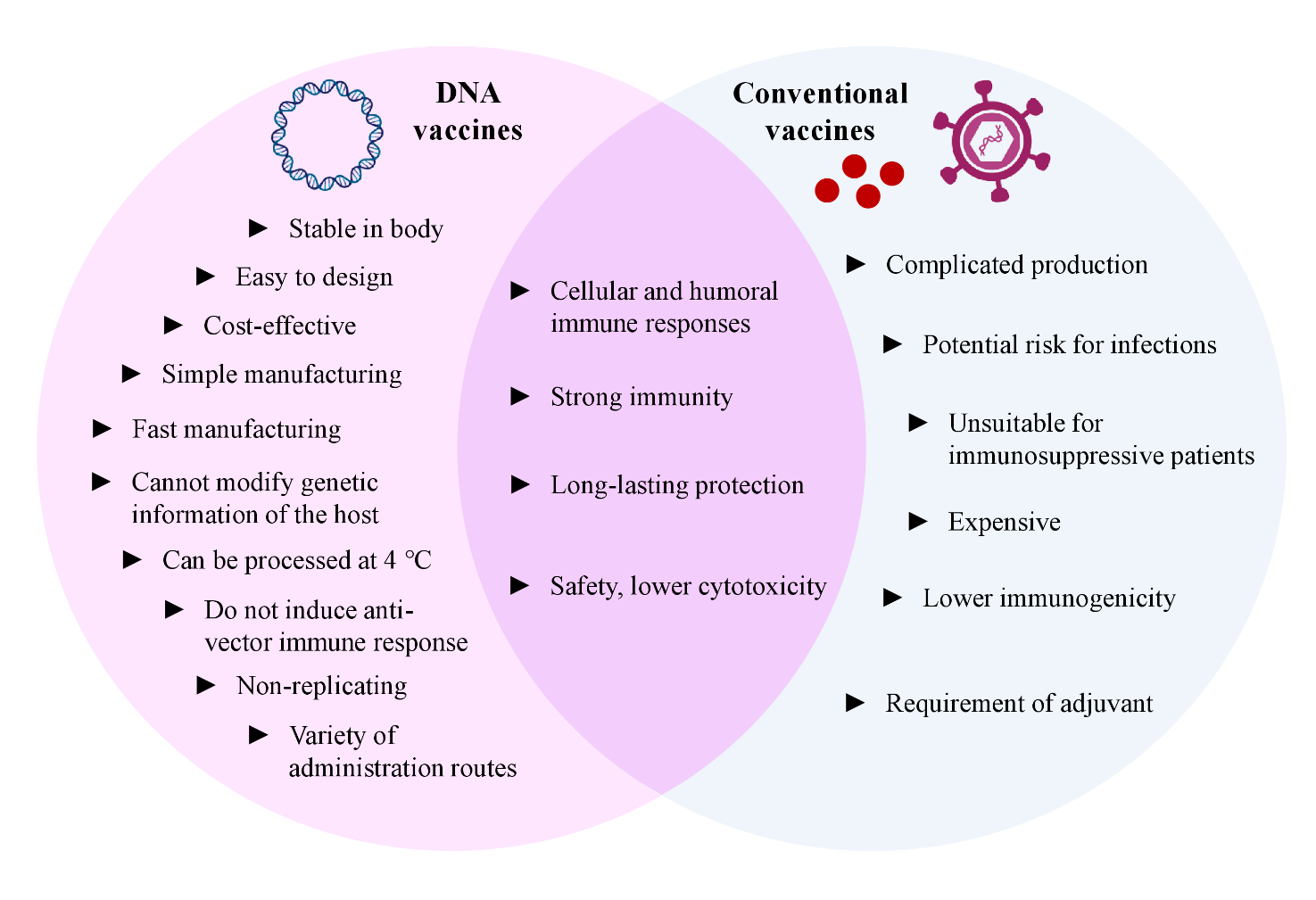
 **Supplementary Figure 4.** DNA vaccines vs conventional vaccines. The overlapping section represents the common features of DNA and conventional immunization approaches.

## Supplementary Tables

**Supplementary Table 1.** Worldwide human population coverage of each epitope.

| Epitope | Population coverage (worldwide) |
| --- | --- |
| APILLPSSTAPVLKP | 69.22% |
| DQILQLVKGFERFQK | 82.46% |
| MLMIGNYFSGVLIAG | 59.76% |
| HRKVVSSTTQYDHKE | 30.53% |
| HSDYKSFEDAKANCA | 48.56% |
| DSGYHSLDPNAVCET | 36.12% |
| MCTVSDYVSELYDKP | 18.41% |
| ETNDLVTNVY | 17.34% |
| TLKDLMSSV | 39.25% |
| LPSSTAPVL | 12.78% |
| YQRQLREQL | 45.51% |
| HTNHSDISM | 17.34% |
| FILGIIITV | 39.25% |
| SPTRTWKVL | 12.78% |
| SPTRTWKV | 10.55% |
| DSDVSQEVRKY | 17.34% |
| YQDSDVSQEV | 39.25% |
| LPNKSDVL | 12.78% |
| NKRKRVIGL | 10.55% |
| VSDYVSELY | 17.34% |
| FLISIIVLV | 39.25% |
| KPLYEVNSTM | 47.01% |
| DQYKFHKL | 10.55% |
| Epitope set | 95.21% |

**Supplementary Table 2.** Tools and their links that were used in this study.

| Server/Tool Name | Links |
| --- | --- |
| Jalview | <https://www.jalview.org/> |
| NetMHCpan v4.1 | <https://services.healthtech.dtu.dk/services/NetMHCpan-4.1/> |
| NetMHCIIpan v4.0 | <https://services.healthtech.dtu.dk/services/NetMHCIIpan-4.0/> |
| BepiPred v2.0 | <http://tools.iedb.org/bcell/> |
| AllerCatPro | <https://allercatpro.bii.a-star.edu.sg/> |
| Vaxijen v2.0 | <http://www.ddg-pharmfac.net/vaxijen/VaxiJen/VaxiJen.html> |
| ProtParam | <https://web.expasy.org/protparam/> |
| RoseTTAFold | <http://robetta.bakerlab.org/> |
| GalaxyRefine | <https://galaxy.seoklab.org/cgi-bin/submit.cgi?type=REFINE> |
| FG-MD | <https://zhanggroup.org/FG-MD/> |
| PyMod (Ramachandran plot) | <https://pymolwiki.org/index.php/PyMod> |
| SAVES v6.0 (ERRAT) | <https://saves.mbi.ucla.edu/> |
| ElliPro | <http://tools.iedb.org/ellipro/> |
| C-ImmSim | <https://kraken.iac.rm.cnr.it/C-IMMSIM/> |
| ClusPro | <https://cluspro.org/> |
| DeepSoluE | http://lab.malab.cn/~wangchao/softs/DeepSoluE/ |
